# Expression profiles of p53/p73, NME and GLI families in metastatic melanoma tissue and cell lines

**DOI:** 10.1038/s41598-019-48882-y

**Published:** 2019-08-28

**Authors:** Petar Ozretić, Nikolina Hanžić, Bastien Proust, Maja Sabol, Diana Trnski, Martina Radić, Vesna Musani, Yari Ciribilli, Ivan Milas, Zvonimir Puljiz, Maja Herak Bosnar, Sonja Levanat, Neda Slade

**Affiliations:** 1 Division of Molecular Medicine, Ruđer Bošković Institute, Bijenička cesta 54, HR-10000 Zagreb, Croatia; 2 Department of Cellular, Computational and Integrative Biology (CIBIO), University of Trento, Via Sommarive 9, Povo (Trento), IT-38123 Italy; 3 Sestre milosrdnice University Hospital Center, Vinogradska cesta 29, HR-10000 Zagreb, Croatia

**Keywords:** Melanoma, Molecular medicine

## Abstract

Unlike other tumours, *TP53* is rarely mutated in melanoma; however, it fails to function as a tumour suppressor. We assume that its functions might be altered through interactions with several families of proteins, including p53/p73, NME and GLI. To elucidate the potential interplay among these families we analysed the expression profiles of aforementioned genes and proteins in a panel of melanoma cell lines, metastatic melanoma specimens and healthy corresponding tissue. Using qPCR a higher level of *NME1* gene expression and lower levels of Δ40p53β, ΔNp73, *GLI1*, *GLI2* and *PTCH1* were observed in tumour samples compared to healthy tissue. Protein expression of Δ133p53α, Δ160p53α and ΔNp73α isoforms, NME1 and NME2, and N′ΔGLI1, GLI1FL, GLI2ΔN isoforms was elevated in tumour tissue, whereas ∆Np73β was downregulated. The results in melanoma cell lines, in general, support these findings. In addition, we correlated expression profiles with clinical features and outcome. Higher Δ133p53β and p53α mRNA and both *GLI1* mRNA and GLI3R protein expression had a negative impact on the overall survival. Shorter overall survival was also connected with lower p53β and *NME1* gene expression levels. In conclusion, all examined genes may have implications in melanoma development and functional inactivity of *TP53*.

## Introduction

Malignant melanoma remains the most aggressive and treatment-resistant form of skin cancer with increasing incidence^1^. Although recent advances in melanoma therapy improve the overall patient survival, they are still hampered by rapid and pervasive treatment resistance. Thus, novel molecular approaches that would contribute to a better understanding of the disease should be deployed.

p53 plays a substantial role in the response to stress by coordinating diverse signalling pathways, thereby preventing the tumour formation. Unlike other cancers, in metastatic melanoma *TP53* gene is relatively rarely mutated. However, it fails to function as a tumour suppressor and reduced levels of p53 contribute to aggressiveness and resistance to therapy^2^. Several diverse mechanisms of p53 inactivation in melanomagenesis have been proposed. The most common mechanisms include mutations of cyclin-dependent kinase inhibitor *CDKN2A* (encoding for both p16INK4A and p14ARF, which inactivates MDM2) and *MDM2* (negative regulator of p53) overexpression, activation of iASPP (inhibitor of apoptosis stimulating protein of p53) or silencing of the *TP53* gene by epigenetic mechanisms^2–5^. However, the later phenomenon has not been fully understood.

**Figure 1:**
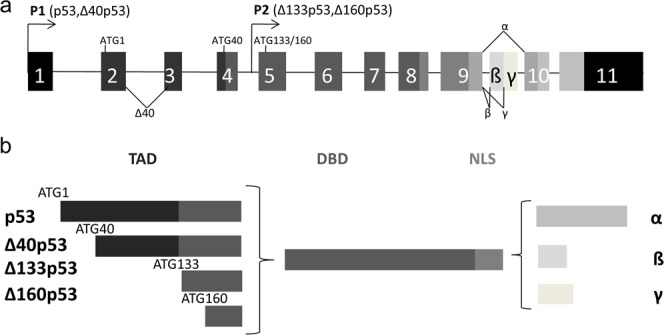
Scheme of the *TP53* gene (**a**) and the protein isoforms (**b**). The colour of the protein domains matches the corresponding exon. Black boxes represent noncoding sequences, whereas coding sequences are in grayscale. The *TP53* gene is composed of 11 classical exons and alternative exons (9 β and γ). Furthermore, it contains an internal promoter (P2), alternative splice variants (Δ40, β, γ) and internal initiation of translation sites (ATG1, ATG40, ATG133, ATG160). The p53 protein is composed of several domains - transactivation domain (TAD, which actually include two different domains, TAD1 and TAD2), the DNA-binding domain (DBD) and the nuclear localization signal (NLS). Twelve protein isoforms with different N- and C-termini are encoded by the human *TP53* gene – alternative splicing of human intron 2 gives rise to Δ40p53 (truncated transactivation domain, TAD) and intron 9 to α, β and γ isoforms; the usage of the alternative promoter produces Δ133p53 and Δ160p53 (both lacking the entire TAD), while alternative translation initiation site produces Δ40p53 and Δ160p53.

The p53 family comprises of p53, p73 and p63. Transcription from alternative promoters, alternative splicing and diverse translation initiation sites contribute to the family complexity^6,7^, and twelve protein isoforms with different N- and C-termini are encoded by the single *TP53* gene (Fig. 1)^8^. The diversity in structure leads to diversity in subcellular localization, and consequently in various biochemical/biological activities which are cell-type dependent. Finally, p53-mediated cell response is the sum of the activities which results from co-expressed p53 isoforms^8^. Currently, the evidence explaining the involvement of p53 isoforms in tumour formation is still limited.

Likewise, two main groups of p73 isoforms with different N-termini are produced: the TAp73 isoforms with the entire TAD and the N-terminally truncated isoforms, ΔNp73, lacking TAD and acting, mainly, as dominant-negative inhibitors towards p53 and TAp73^9^. In contrast to p53, p73 is essentially never mutated in cancer, but it is overexpressed^6,10^.

Most of the p53 family isoforms possess the ability to interact between themselves forming heterotetramers and compete for DNA binding, so the overall activity of p53/p73 proteins is a result of the ratio between the different isoforms. Thus, in melanoma it has been found that p63 interacts with p53 influencing its tumour suppressive role^11^, while the Δ40p53 isoform forms heterotetramers with p53 modifying downstream p53 target genes and promoting apoptosis over cell cycle arrest^12^. However, we assume that p53 function in metastatic melanoma might be altered through interactions with some other proteins, like NME and GLI families of proteins.

The *NME* (NM23, NDPK, Awd) gene/protein family consists of ten members named after the first one identified, NME1/NM23-H1/NDPKA^13^, which is considered to be a metastasis suppressor gene. Besides its enzymatic activity (nucleoside-diphosphate kinase), the NME proteins have been assigned several additional biochemical functions such as transcription regulators, protein kinases and DNases^14^. A connection between NME expression and melanoma formation or metastasis has been described in several reports^15–17^. Although the investigations were primarily focused on *NME1* as a primary metastasis suppressor, some data on *NME2* in murine and human melanoma are also available^18–20^. Lately, several independent studies suggest there is either a direct or indirect connection between p53 and NME gene/protein family members^21–23^.

The Hedgehog-GLI (HH-GLI) signalling pathway plays a vital role during embryonic development, stem and progenitor cell maintenance, and carcinogenesis. Binding of the Hedgehog (HH) ligand to the receptor Patched (PTCH1) triggers the cascade leading to the activation of transcription factors GLI1, GLI2 and GLI3. They regulate cell proliferation, cell cycle regulation, adhesion, epithelial-mesenchymal transition, self-renewal and pathway autoregulation (*PTCH1* and *GLI1* genes)^24^. GLI1 is the exclusive activator, while GLI2 and GLI3 are cleaved into repressor forms when the HH signal is not present^25^. *PTCH1*, *GLI1* and *GLI2* overexpressions have been associated with melanoma progression and invasive and metastatic phenotype^26,27^. The HH-GLI pathway is also governed by non-HH signalling pathways by interactions with other pathways, including p53^28–30^. p53 negatively inhibits GLI1-driven neural stem cell self-renewal, cell proliferation and tumour growth and, in turn, GLI1 represses p53^31,32^. The balance between p53 and GLI1 activities appears to be critical: loss of p53 in cancer could enable activities of GLI1 and GLI2^32,33^, while repression of GLI3 activity potentiates p53-dependent cell growth inhibition in colon cancer cells^34^.

The aim of this work is to study the potential interplay between these three pathways involved in melanoma development and progression. To gain this knowledge, it was necessary to determine the expression pattern of a range of isoforms of the above mentioned proteins, which have not been systematically investigated in melanoma so far. In all the studies performed so far on metastatic melanoma, p53 isoforms’ expression was determined in cell lines, not in surgical tissue samples, while GLI isoforms have not been examined at all. Therefore, we investigated the expression of three groups of genes and proteins and their correlation to clinicopathological characteristics.

## Results and Discussion

### Gene expression in metastatic melanoma tissue samples and cell lines

**Figure 2:**
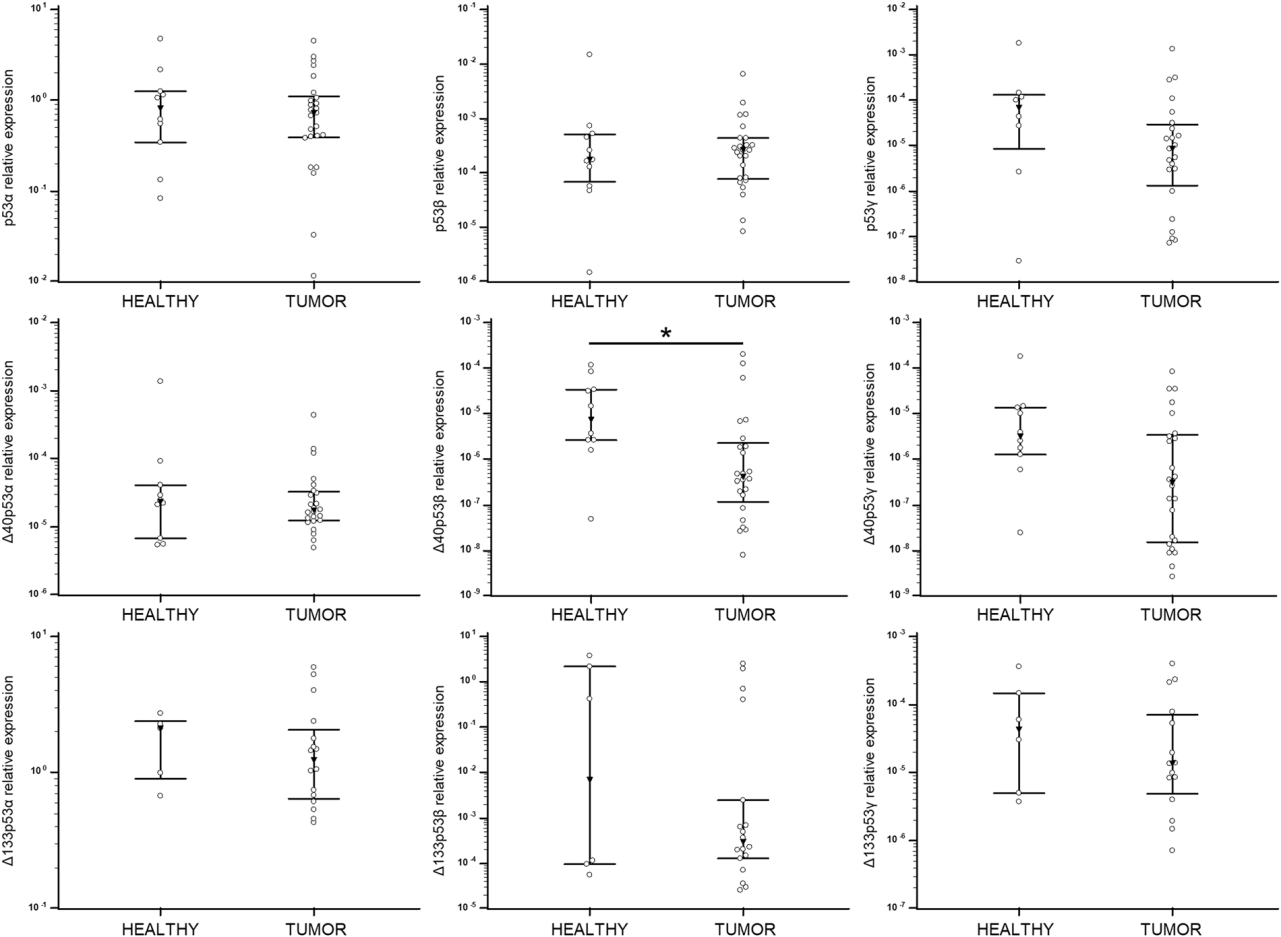
Relative expression levels of *TP53* isoforms analysed by pre-amplification followed by qPCR using SYBR Green dye. The expression was normalized to the expression level of total *TP53*. Results are presented in a log scale, the bars represent interquartile range and the black triangle represents median value. *Denotes *p* < 0.05.

Gene expression analysis was performed on 32 tumour tissue samples and 19 healthy skin samples from the same patients. The *TP53* isoforms were pre-amplified in two separate pre-amplification reactions, giving a “long” and “short” template for quantitative real-time PCR (qPCR) amplification and analysis. The mean relative expression of “long” *TP53* isoforms in tumour tissue, ranked from the highest to the lowest, was as follows: p53α > p53β > p53ɣ > ∆40p53α > ∆40p53β > ∆40p53ɣ. Ranking of the mean relative expression of “short” *TP53* isoforms was: ∆133p53α > ∆133p53β > ∆133p53ɣ. Only one out of nine *TP53* isoforms was significantly downregulated in tumour tissue (∆40p53β, *p* = 0.017) (Fig. 2). A similar trend was observed for the ∆40p53ɣ isoform but without statistical significance (*p* = 0.054). The expression levels of the studied genes were confirmed using a panel of eight melanoma cell lines. We have observed almost a perfect match of the mean gene expression levels in cell lines and metastatic melanoma tissues with a small deviation of ∆40p53β and ∆40p53ɣ and the shortest, Δ133p53γ. The role of ∆40p53β was not particularly studied so far. However, based on obtained data, it has to be taken into account.

Previous clinical studies have reported the expression of *TP53* isoforms in several tumour types, confirming that small molecular weight p53 isoforms might play an important role in tumorigenesis^12,35–40^. Avery-Kiejda and collaborators described that p53β and ∆40p53 mRNAs were expressed at higher levels than p53α in most melanoma cell lines examined, compared to fibroblasts and melanocytes, suggesting that their expression may play a role in melanoma development^41^. However, none of these aforementioned studies applied a sophisticated approach in which qPCR was performed as a nested reaction following initial RT-PCR amplification.

**Figure 3:**
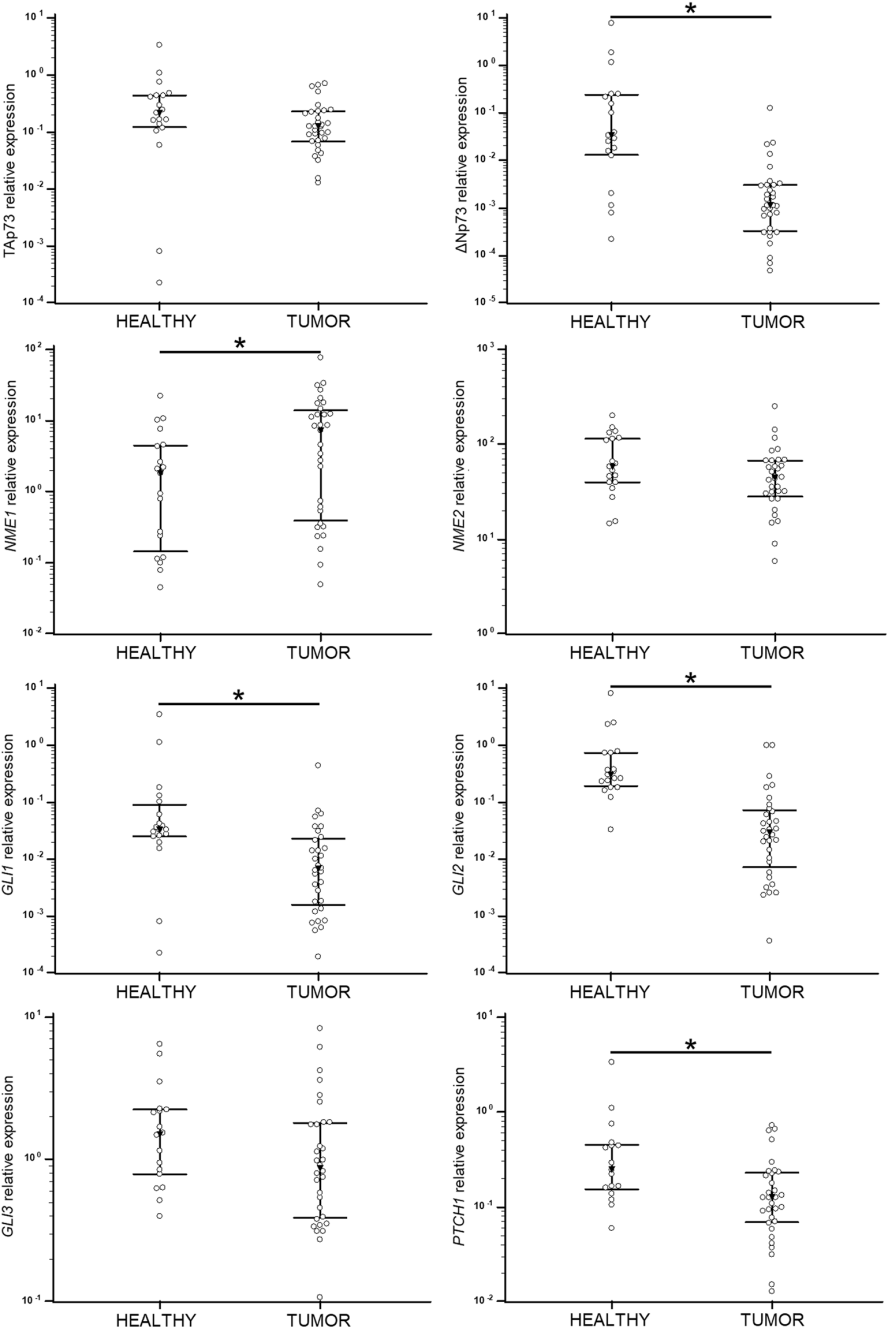
Relative expression of genes analysed by TaqMan assays. The expression was normalized to the expression level of *GUSB* and *TBP* (2^−ΔCt^). Results are presented in a log scale, the bars represent interquartile range and black triangle represents median value. *Denotes *p* < 0.05.

The remaining genes were analysed using standard TaqMan assays. We were able to determine the gene expression of two cancer-relevant *TP73* isoforms, TAp73 and ∆Np73. Unexpectedly, the expression of full length isoform TAp73 was higher than ∆Np73. Further, ∆Np73 expression was significantly downregulated in metastatic melanoma tissue (*p* < 0.0001) (Fig. 3). There is a paucity of studies that have analysed the p73 isoforms’ gene expression in melanoma. The only detailed study of the expression and effect of particular p73 isoforms in metastatic melanoma showed overexpression of TAp73, Ex2p73 and Ex2/3p73 (spliced transcripts derived from the first promoter), whereas ΔNp73 was the predominant isoform in benign nevi^42^, which is in line with our findings.

In our study a significant downregulation of *GLI1* (*p* = 0.001), *GLI2* (*p* < 0.0001) and *PTCH1* (*p* = 0.006) in tumour tissue was observed. Several studies have shown upregulation of HH-GLI signalling in melanoma. A study on clinical samples showed that melanoma cells express *SHH*, *GLI1* and *PTCH1* mRNA, but the surrounding stroma does not^26^. Another study showed upregulation of *SMO*, *GLI2* and *PTCH1* mRNA in a subset of melanoma cell lines^43^. *GLI2* was found to be associated with invasive and metastatic phenotype in melanoma. Tumours with high *GLI2* expression metastasize to the bone more quickly than tumours with low *GLI2* expression^27^.

Several groups of authors analysed the correlation between the RNA and protein levels of NME and their metastatic potential with heterogenous results^15–17,44,45^. Although there has been evidence that the *NME1* RNA levels can be elevated while the protein levels are low due to protein degradation in invasive melanoma cells^46^, in our experiments NME1, as well as NME2, was significantly up-regulated on both, the RNA and protein level in tumours compared to healthy tissue (p = 0.041) (Figs 3, 5 and Supplementary Fig. S1).

**Figure 4:**
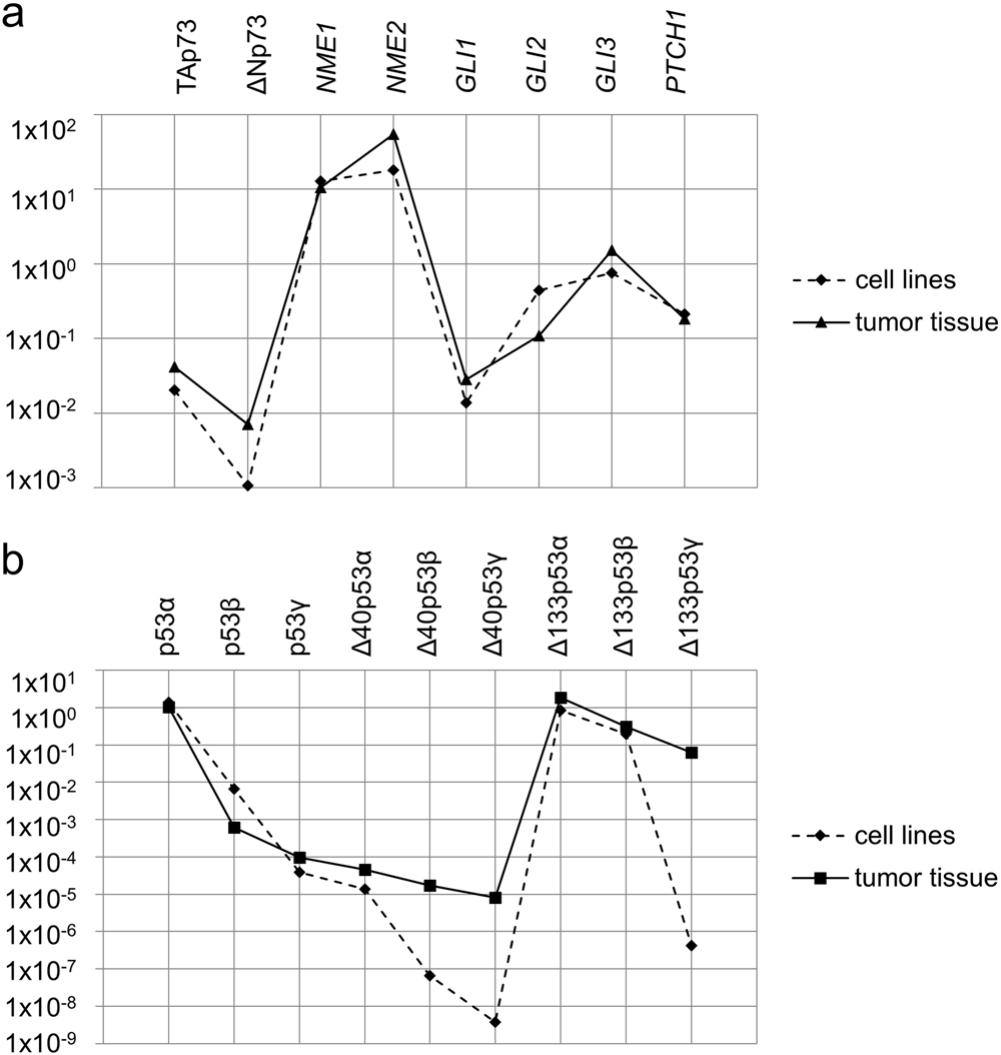
Comparison of relative expression values for tumour samples (solid line) and melanoma cell lines (dashed line) normalized to the expression level of *GUSB* and *TBP*. (**a**) Mean expression values of *TP53* isoforms analysed by pre-amplification and SYBR Green RT-qPCR. (**b**) Mean expression of other tested genes analysed by TaqMan assays.

**Figure 5:**
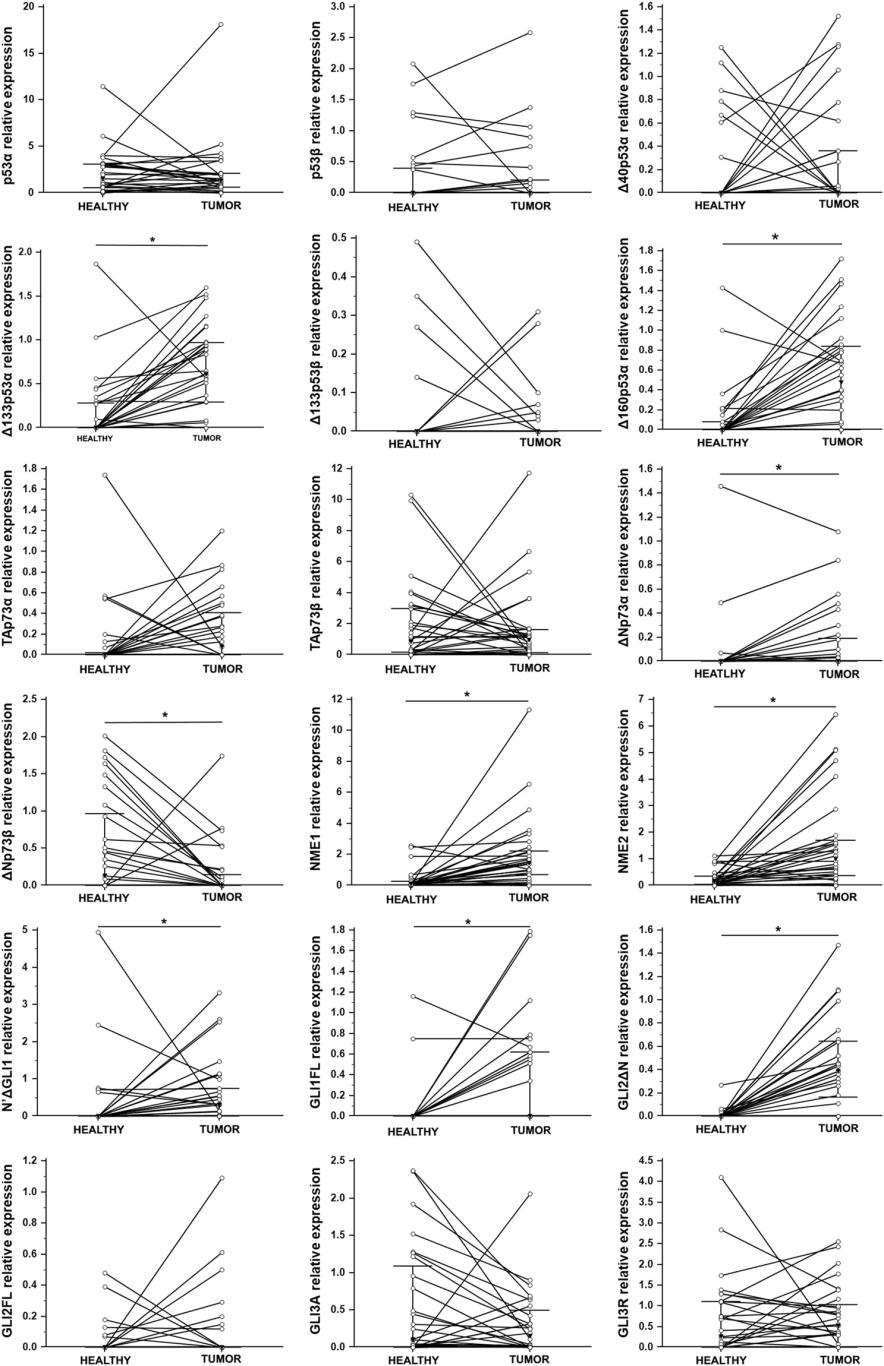
Expression of p53, p73, NME and GLI proteins. Proteins were analysed by PAGE, Western blot and densitometry with samples normalized to actin staining, followed by Wilcoxon paired sample analysis. P-values for proteins with significant difference in expression between tumour and healthy tissue are marked in bold. *Denotes *p* < 0.05.

The mean expression of *TP73*, *NME* and HH-GLI signalling pathway genes in a panel of eight melanoma cell lines almost completely match the mean expression in metastatic melanoma tissue (Fig. 4a). The expression levels of *TP53* isoforms in melanoma cell lines showed some differences. It can be observed that ∆40p53 isoforms, particularly the shortest Δ133p53γ, showed lower expression levels in cell lines compared to tumour tissue samples (Fig. 4b). *TP53* isoforms were not expressed at all in LM6 (4405 P) cell line probably due to the lack of *TP53* locus^47^.

### Protein expression in metastatic melanoma tissue samples and cell lines

**Figure 6:**
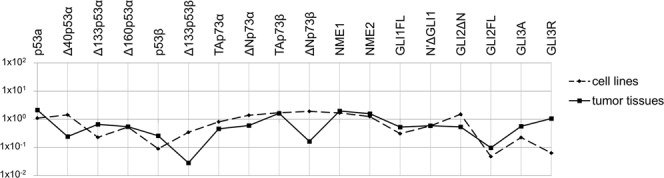
Comparison of mean normalized protein expression values for tumour samples (solid line) and melanoma cell lines (dashed line). Protein expression was determined by densitometry and normalized to actin.

In 30 paired tumour and healthy skin tissue samples we quantified the expression of 18 proteins in total: six p53 isoforms differing by both N- and C-termini (p53α, p53β, ∆40p53α, ∆133p53α, ∆133p53β and ∆160p53α); four p73 isoforms (TAp73α, TAp73β, ∆Np73α and ∆Np73β); NME1 and NME2; two GLI1 isoforms (N′ΔGLI1 and GLI1FL, molecular weights 130 and 160 kDa, respectively); two GLI2 isoforms (GLI2ΔN and GLI2FL, molecular weights 133 and 250 kDa, respectively), GLI3 activator (GLI3A, 190 kDa) and GLI3 repressor (GLI3R, 83 kDa). Summary of the results comprising the number and percentage of positive healthy and tumour samples is shown in Supplementary Table S1. When compared with healthy tissue, eight proteins showed statistically significant higher expression in tumours: both GLI1 isoforms (N′ΔGLI1, *p* = 0.030; GLI1FL, *p* = 0.002), GLI2ΔN isoform (*p* < 0.0001), both NME1 and NME2 (*p* = 0.0001 and *p* < 0.0001, respectively), ∆Np73α (*p* = 0.020) and two p53 isoforms - ∆133p53α and ∆160p53α (*p* = 0.0002 and *p* = 0.001, respectively). Conversely, the expression of ∆Np73β isoform was significantly downregulated in tumour tissue (*p* = 0.005) (Fig. 5). Further, the mean expression levels in a panel of eight melanoma cell lines were investigated (Supplementary Fig. S1) and when compared with metastatic melanoma tissue, almost perfect match of mean expression levels was observed with exception of Δ133p53β, ΔNp73 and GLI3R (Fig. 6).

Due to the different site of the translation initiation, the presence of all known protein isoforms can be determined only by Western blot using specific antibodies that recognize a particular set of isoforms and p53/p73 standards (cells transfected with the specific isoform) helped to identify every single isoform (Supplementary Fig. S1). Previous studies reported wild type p53 overexpression in melanoma cell lines and tissue samples^48,49^. However, in these studies the authors used immunohistochemical analysis in which it was impossible to distinguish the specific isoforms. Therefore, our study is the first detailed study of p53 isoform expression in metastatic melanoma tissue.

Using this approach, ΔNp73α protein was significantly more expressed in the metastatic melanoma than in healthy tissue as compared to gene expression analysis. These inconsistencies between gene and protein expression are not unusual, since post-transcriptional regulation plays an important role in modification of proteins. Also, the experimental approach we used enabled us to distinguish the isoforms more precisely. Overexpression of total p73 in invasive cutaneous melanoma was recently shown using immunohistochemistry^50^. Overexpression of ΔNp73 is reasonable considering that ΔNp73 drives migration and invasion of nonmetastatic melanoma cells^51^.

As mentioned earlier, our results show elevated NME1 and NME2 mRNA and protein levels in metastatic melanoma although we would expect them to drop in metastasis due to the proposed role of NME as a metastasis suppressor. However, the mechanism by which NME contributes to tumour progression seems to be rather complex and does not rely solely on the rate of their expression. It has been reported that the NME1 content significantly varies in different cell populations within a tumour tissue sample. As an example, it has been suggested by Martinez and co-workers that NME1 could be less expressed in the invasion front compared to other cellular populations within the tumour mass^52^ which would probably be possible to detect immunohistochemically but not using Western blot, as was the case in our study.

Again, we found a discrepancy between gene and protein expression regarding HH-GLI pathway components. In recent years it has become clear that the GLI code is more complex than previously thought, with five known isoforms of GLI1, five of GLI2 (two of which with repressive activity), and two of GLI3, the GLI3A and GLI3R (reviewed in^53^) which can be distinguished only by Western blot analysis. Interestingly, Roessler and collaborators have demonstrated that GLI2ΔN is up to 30-fold more potent than the full length GLI2^54^. GLI2ΔN can induce genomic instability by interfering with the cell cycle, acting through downregulation of mitosis regulators 14-3-3σ and p21^WAF1/CIP1^ ^55^.

### Correlation of gene and protein expression

Several *TP53* isoforms were found to be correlated on the level of gene expression. A very strong correlation (ρ > 0.8) was found between Δ40p53γ and Δ40p53β, Δ133p53γ and Δ133p53α, and Δ133p53γ and Δ133p53β isoforms. A strong correlation (0.8 > ρ > 0.6) was found between Δ133p53β and Δ133p53α, TAp73 and Δ133p53β and a moderate correlation (0.6 > ρ > 0.4) between p53γ and p53α, Δ40p53β and Δ40p53α. For other genes tested, a strong correlation was determined for *GLI1* and Δ133p53α, and *GLI1* and *GLI2*, a moderate correlation for *NME1* and *NME2*, *GLI2* and *NME2*, *PTCH1* and *NME2*, and *PTCH1* and ΔNp73. A weak correlation (ρ < 0.4) was found between *GLI2* and TAp73, and *GLI3* and ΔNp73 (Supplementary Table S2).

At the protein level, a strong correlation was detected between TAp73β and TAp73α, and TAp73β and p53α. The remaining correlations between p53/p73 isoforms were mostly moderate (p53α with Δ133p53β, Δ160p53α, TAp73α and ΔNp73α; Δ133p53α with Δ133p53β, Δ160p53α with TAp73β; Δ160p53α with TAp73α; TAp73α with TAp73β and ΔNp73α). Interestingly, the expression of Δ133p53β was only negatively correlated with other isoforms.

NME1 protein was weakly correlated with ΔNp73α, but strongly with NME2 and GLI2ΔN. A mutual correlation was found between the two GLI1 isoforms, while GLI1FL isoform was negatively correlated with GLI2FL isoform. Unlike GLI1, where both isoforms were similarly correlated with other proteins, for the correlations between GLI3A and GLI3R the correlations were completely different: GLI3A correlated weakly with TAp73α and moderately with N′ΔGLI1, while GLI3R correlated strongly with p53α, moderately with TAp73β and weakly with GLI3A (Supplementary Table S3).

Accordingly, a strong correlation was found primarily between the family members, which is somehow expected because isoforms often share common transcriptional regulation. However, observed correlations between members of different families, e.g. negative correlation between *GLI1* and *TP53* isoforms or positive correlations between either *TP73* isoforms or *NME2* and HH-GLI pathway genes, are much more interesting since they can indicate a potential interplay among those families of genes and proteins.

There was almost no correlation between gene and protein expression, with *NME1* gene being the only exception showing a moderate correlation between gene and protein expression (ρ = 0.43, *p* = 0.031) (data not shown). We assume this is a consequence of using different translation initiation sites and numerous translational modifications happening within p53 and GLI families of proteins.

### Determination of *BRAF* and *TP53* mutation status

Twenty out of 38 samples (52.6%) were positive for *BRAF* mutation. The most frequent *BRAF* mutation found in melanoma, c.1799T > A (p.V600E), was found in 19 cases (95%), while c.1798_1799delGTinsAA (p.V600K) was found in the remaining mutation positive sample. This is in line with the available literature^56^.

Conversely, only 3 out of 30 samples were positive for *TP53* mutations (Supplementary Table S4). This observation is consistent with previously studies demonstrating the mutation of *TP53* locus as a rare event during melanomagenesis^56^; between 15–20% according to the cBioPortal online tool of TCGA Consortium (http://www.cbioportal.org/).

### Levels of gene/protein expression and their relation to clinicopathological characteristics

**Figure 7:**
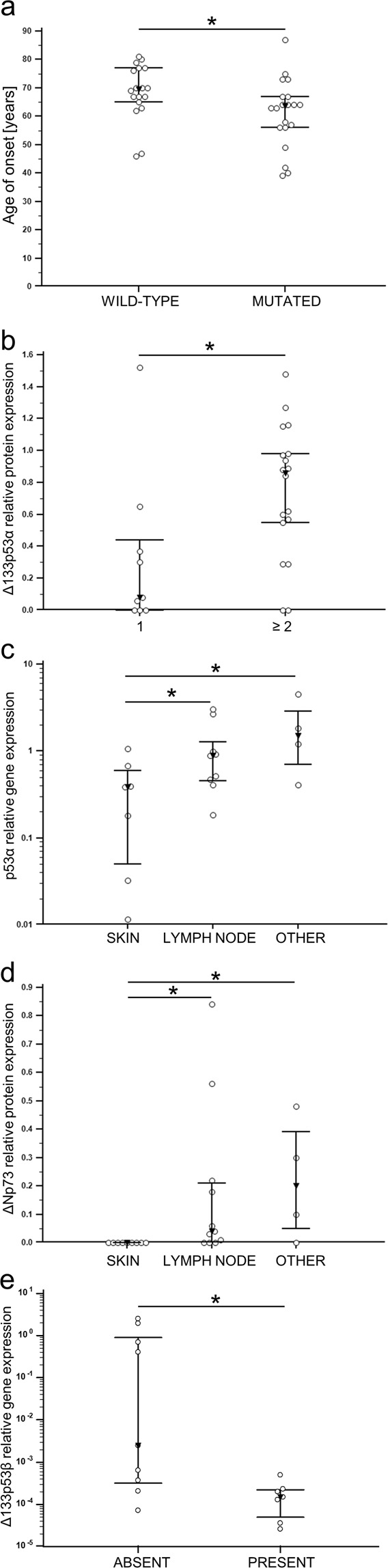
Association of patients’ characteristics and gene/protein expression. (**a**) BRAF mutation status is associated with age of onset (*p* = 0.019). (**b**) Occurrence of previous metastases is associated with Δ133p53α protein expression (*p* = 0.039). (**c**) Location of sampled tissue is associated with the expression of p53α gene (*p* = 0.033) and (**d**) ΔNp73α protein (*p* = 0.039). (**e**) Development of subsequent metastasis is associated with Δ133p53β gene expression (*p* = 0.013). Black rectangle represents the median value and bars represent the interquartile range. *Denotes *p* < 0.05.

We analysed the association of various patients’ characteristics mutually and in relation to gene/protein expression. It was observed that *BRAF* mutation-positive patients tend to develop metastases at a younger age (median 63.5 years vs. 69.5 years, *p* = 0.019) (Fig. 7a). This was in accordance with Ekedahl and colleagues who in a clinic-based metastatic melanoma cohort found that BRAF mutation was associated with a younger age at primary diagnosis^57^. In tumour tissue from *BRAF* mutation-positive patients has been noticed a trend of higher *GLI3* expression (*p* = 0.058) (data not shown). Although it was found that the expression of GLI1 is higher in human primary melanoma harbouring BRAF p.V600E mutation^58^, we did not detect such correlation in our studies. Interestingly, tumour samples from patients who had two or more previous metastases had significantly higher Δ133p53α protein expression levels compared to those with only one previous metastasis (*p* = 0.039) (Fig. 7b). In addition, it was observed that location of a specific metastatic tissue has an impact on the expression of various genes and proteins. For instance, skin metastases had the lowest gene expression of p53α compared to metastases from lymph nodes or other locations (*p* = 0.033) (Fig. 7c). The same was observed for ΔNp73α protein (*p* = 0.039) (Fig. 7d), while the opposite trend (higher expression in skin metastases) was determined for ΔNp73 gene expression (*p* = 0.057) (data not shown). Interestingly, the expression of Δ133p53β gene was significantly lower in tumour samples from patients who developed subsequent metastases (*p* = 0.013) (Fig. 7e).

### Impact of gene/protein expression and clinicopathological characteristics on patients’ survival

**Figure 8:**
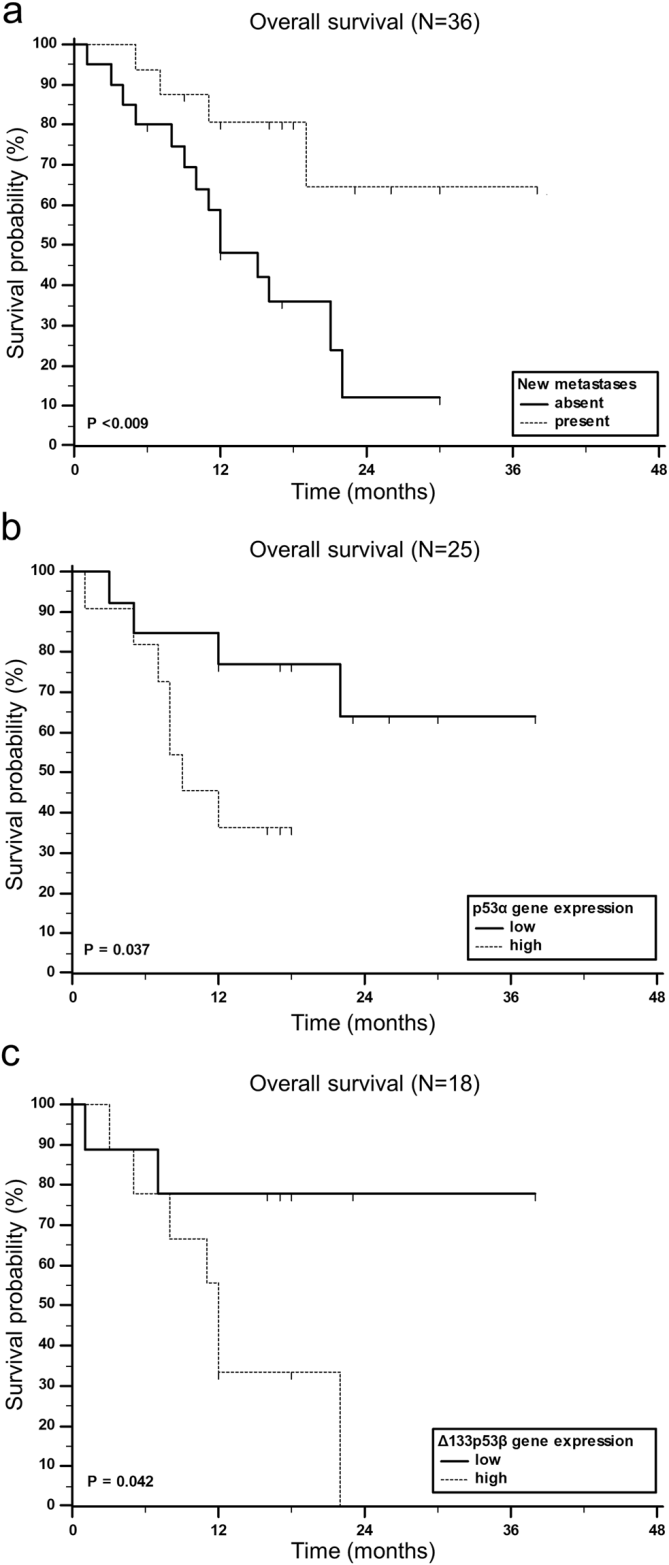
Kaplan-Meier survival curves showing significant impact of clinicopathological characteristic and gene expression levels on overall survival (OS) for metastatic melanoma patients. (**a**) OS according appearance of new metastasis. (**b**) OS according p53α gene expression. (**c**) OS according Δ133p53β gene expression. Tick marks indicate censored cases.

Nineteen patients (50%) died during the follow-up period. The median survival time of our patients was 19 months. To examine the impact of clinicopathological characteristics of patients and gene/protein expression profiles on overall survival (OS), a univariate analysis was performed. Among all patients’ characteristics, only the development of new metastasis has shown to have a significant impact on OS. Interestingly, patients who did not develop subsequent metastases had almost four times higher chances to die compared to those with at least one new metastasis (hazard ratio (HR) 3.88, 95% confidence interval (CI) 1.54–9.78, *p* = 0.009) (Fig. 8a). Although counterintuitive, it could be easily assumed that those patients from the start had a more severe form of disease, and in consequence, did not live long enough to develop new metastases.

Furthermore, patients with high (above median value) p53α gene expression tended to live shorter compared to those with low p53α expression (HR 3.16, 95% CI 0.99–10.09, *p* = 0.037) (Fig. 8b). In addition, patients with high Δ133p53β gene expression had more than four times higher risk to die compared to those with low Δ133p53β expression (HR 4.30, 95% CI 1.16–15.98, *p* = 0.042) (Fig. 8c). This could be connected with patients who did not develop subsequent metastases which have particularly higher Δ133p53β expression (Fig. 7e), and both have a negative impact on OS of patients with metastatic melanoma (Fig. 8a,c). Recently, it was also shown that the elevated expression of Δ133p53β causes tumour cells to spread to other organs regardless of the *TP53* mutation status and increases the risk of cancer recurrence and death in patients with breast cancer^59^. Therefore, it is worthwhile to further explore the prognostic significance of this isoform as a marker for shorter OS in metastatic melanoma patients.

When we dichotomized expression data based on the receiver operating characteristic (ROC) curve analyses, expression of several additional genes and one protein showed a significant impact on OS. Opposite to p53α, patients with low p53β gene expression had shorter OS (HR 5.36, 95% CI 1.52–18.85, p = 0.009) (Supplementary Fig. S2a). Few studies investigated the prognostic value of p53β and reported the association of p53β expression with longer disease-frees survival in *TP53* mutant breast cancer^37^; in cholangiocarcinoma p53β downregulation combined with Δ133p53 upregulation was associated with shorter OS^38^. The tumour suppressive role of p53β is supported with findings which show that p53β enhances both p53 and TAp73-mediated apoptosis^60,61^. Also, p53β was shown to enhance, p53-dependent transcription of p21 and PUMA in melanoma cell line^41^. Low *NME1* expression was also associated with shorter OS in our cohort of metastatic melanoma patients (HR 3.41, 95% CI 1.15–10.11, p = 0.027) (Supplementary Fig. S2b) which is in accordance with its role as a metastasis suppressor. Several groups reported the correlation between low NME1 expression and worse patient prognosis and lower overall survival^15,16,45^. Further support comes from the study where the lowest NME1 protein expression was found in primary skin melanoma samples which metastasized in lymph nodes^62^. However, the same study also showed that there was no difference in patient survival between NME1 high- or low-expressing primary melanomas. On the contrary, in an animal model it was shown that tumours expressing low levels of *NME1* mRNA had significantly reduced survival rates and times^63^. We also observed that patients with high GLI3 expression showed shorter OS compared to patients with lower expression, which was observed for both *GLI3* gene (HR 2.88, 95% CI 1.03–8.02, p = 0.043) (Supplementary Fig. S2c) and GLI3R protein (HR 3.28, 95% CI 1.07–10.00, p = 0.037) (Supplementary Fig. S2d). This is in contrast with one previous study where it was observed that higher expression of *GLI3* mRNA was associated with better survival of metastatic melanoma patients^43^.

Our study again showed that assessment of the association between continuous variables such as gene expression and survival time markedly depends on the method used for binarization of potential biomarker data^64^.

## Conclusion

In summary, in this study we have examined a large number of isoforms of protein families involved in melanoma development, progression and metastasis. Gene expression has proven to be less reliable in the detection of specific isoforms, so protein levels should be examined when discussing protein activity. It has been demonstrated that many isoforms positively or negatively affect the activities of wild type proteins, so specific isoforms should be further examined to establish their role in tumour progression.

## Materials and Methods

### Patients

Metastatic melanoma tissues and matched adjacent healthy skin were obtained from 38 patients (Supplementary Table S4). All patients were treated at the Sestre milosrdnice University Hospital Center and clinical data were available. The study complied with the Helsinki Declaration and was approved by the Ethics Review Committee of Sestre milosrdnice UHC and the Bioethical committee of Ruđer Bošković Institute. Informed consent according to the World Medical Association Declaration of Helsinki was obtained from all patients. The tissues were collected during surgery, frozen immediately in dry ice and stored at −80 °C until extraction. Survival time was measured from the date of surgery to the time of death or the last follow-up observation. The median follow-up of patients at the time of analysis was 16 months (range 1–38 months). The information on mortality was obtained from Croatian National Cancer Registry, Croatian Institute of Public Health.

### RNA extraction, RT and qPCR analysis

RNA was extracted using TRIzol Reagent and/or PureLink RNA Mini spin columns, and reverse transcribed using High Capacity cDNA Reverse Transcription Kit according to the manufacturers’ instructions. qPCR analysis was performed using TaqMan Gene Expression Master Mix and TaqMan Gene Expression Assays (all Thermo Fisher Scientific, MA, USA). Gene expression analyses were performed according to protocol suggested by the manufacturer on 7300 Real-Time PCR System (Thermo Fisher Scientific). To distinguish the different *TP53* isoforms, a nested qPCR approach was used and qPCR was performed on CFX96 Real-Time PCR Detection System (Bio-Rad, CA, USA) using Takyon Low Rox SYBR MasterMix dTTP Blue (Eurogentec, Belgium). All details with primer sequences and positions are provided in Supplementary Materials and Methods, Tables S5 and S6, Fig. S3.

### Protein extraction and western blot analysis

Proteins were extracted from frozen tumour tissues and corresponding healthy skin and separated on SDS-polyacrylamide gels and transferred to nitrocellulose membranes (Merck Millipore, USA). The list of antibodies used and all details are available in Supplementary Materials and Methods. Densitometric quantification of protein levels was determined using ImageJ software (https://imagej.net).

### Extraction of DNA and *BRAF* mutation analysis

DNA was extracted from frozen tumour tissues. Exon 15 of *BRAF* gene was amplified by PCR using F-TTCATGAAGACCTCACAGTAAAAA and R-CCACAAAATGGATCCAGACA primers. PCR products were denatured (HR-1 High-Resolution Melter, Idaho Technology, UT, USA) at 0.2 °C/s ramp rate and melting curves were analysed using HR-1 software. Samples with aberrant melting patterns were sequenced in both directions using Big Dye Terminator 1.1 Cycle Sequencing kit (Thermo Fisher Scientific) on ABI PRISM 310 Genetic Analyzer (Thermo Fisher Scientific).

### *TP53* mutation analysis

Using the RNA extracted and converted into cDNA as described above, we performed the well-established yeast functional assay (FASAY) to determine the *TP53* status in melanoma patients. The *TP53* coding sequence was amplified with a two-step nested PCR using Go-Taq G2 Master Mix (Promega). More details are available in Supplementary Materials and Methods.

### Cell culture

A panel of melanoma cell lines (A375M, CHL1, LM6 (4405 P), Mel224, Mel501, Mel505, WM793B and WM983B) was kindly provided by Dr. Bergamaschi (Barts and The London School of Medicine and Dentistry, London, UK). The cells were grown in DMEM (Sigma Aldrich, USA) or RPMI 1640 (Lonza, Switzerland) supplemented with 10% FBS, 1 mM sodium pyruvate (both Thermo Fisher Scientific), 1% streptomycin - penicillin, and 2 mM L-glutamine (both Sigma Aldrich) and maintained at 37 °C with 5% CO_2_. All cell lines were tested to be mycoplasma free.

### Statistical analysis

Is provided in Supplementary Materials and Methods.

## Supplementary information

Supplementary Information

Supplementary Dataset 1

## Data Availability

All patients’ data including clinical characteristics and the results of gene and protein expressions are available as Supplementary Data file.
