# Supplementary material for: Expression profiles of p53/p73, NME and GLI families in metastatic melanoma tissue and cell lines
Source: Sci Rep. 2019 Aug 28;9:12470. doi: 10.1038/s41598-019-48882-y (PMC6713730; doi:10.1038/s41598-019-48882-y)
Supplement: Supplementary file 1 — Supplementary Information [file 41598_2019_48882_MOESM1_ESM.pdf]

Supplementary Information

Expression profiles of p53/p73, NME and GLI families in metastatic melanoma tissue and cell lines

Authors: Petar Ozretić, Nikolina Hanžić, Bastien Proust, Maja Sabol, Diana Trnski, Martina Radić, Vesna Musani, Yari Ciribilli, Ivan Milas, Zvonimir Puljiz, Maja Herak Bosnar, Sonja Levanat, Neda Slade

Corresponding author: Neda Slade, Division of Molecular Medicine, Ruđer Bošković Institute, Bijenička 54, 10000 Zagreb, Croatia; Tel +38514560926; E-mail: slade@irb.hr

Supplementary Figures S1-S3

Supplementary Tables S1-S6

Supplementary Materials and Methods

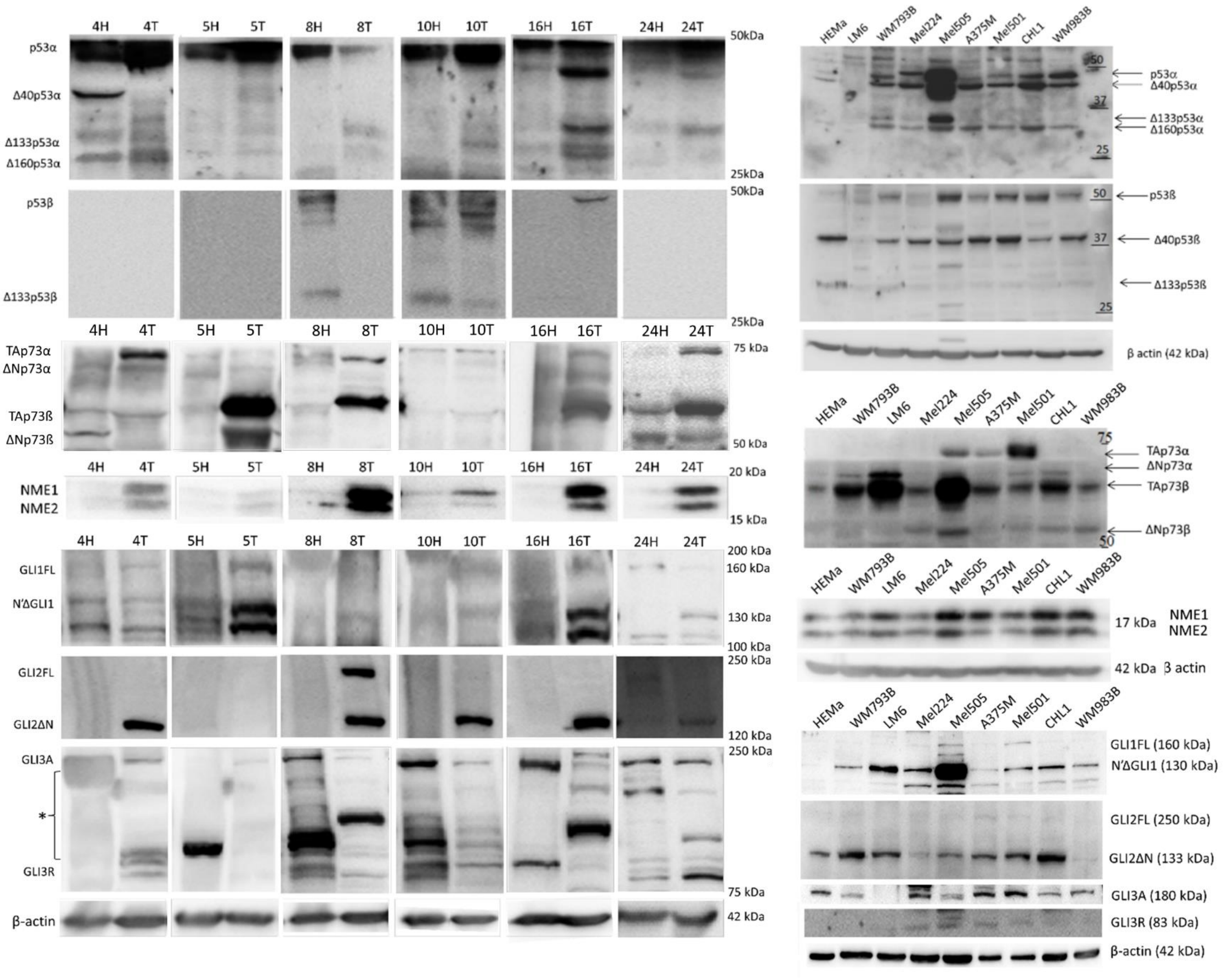

Supplementary Figure S1: Representative western blots of p53/p73/NME/GLI-families of proteins in metastatic melanoma and healthy skin tissue samples (left panes) and melanoma cell lines (right panes). H - healthy tissue, T - tumor tissue, * - nonspecific bands.

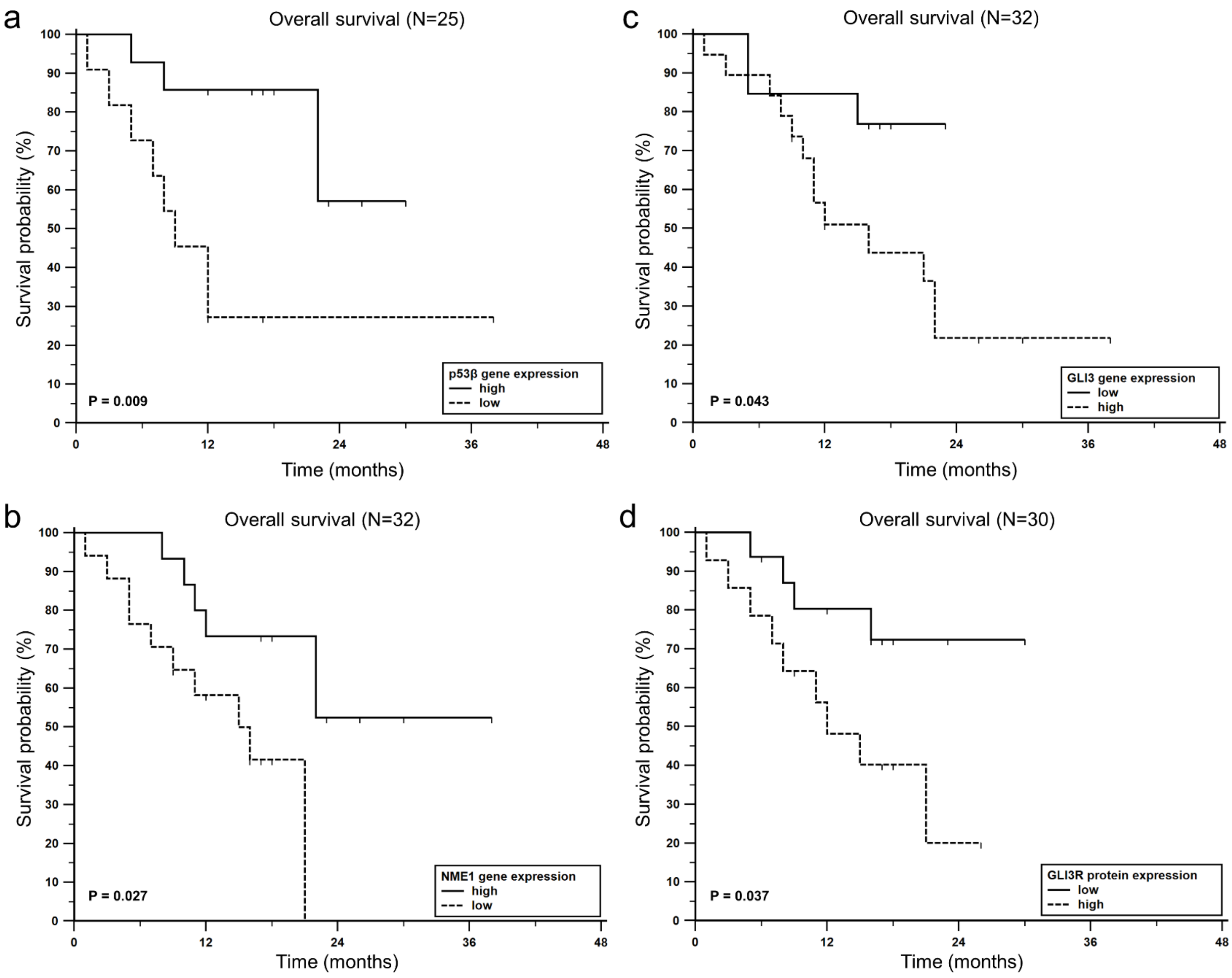

Supplementary Figure S2: Kaplan-Meier survival curves showing significant impact of gene and protein expression levels on overall survival (OS) for metastatic melanoma patients. Expression data was dichotomized into 'low' and 'high' based on the receiver operating characteristic (ROC) curve analyses. (**a**) OS according p53β gene expression. (**b**) OS according *NME1* gene expression. (**c**) OS according *GLI3* gene expression. (**d**) OS according GLI3R protein expression. Tick marks indicate censored cases.

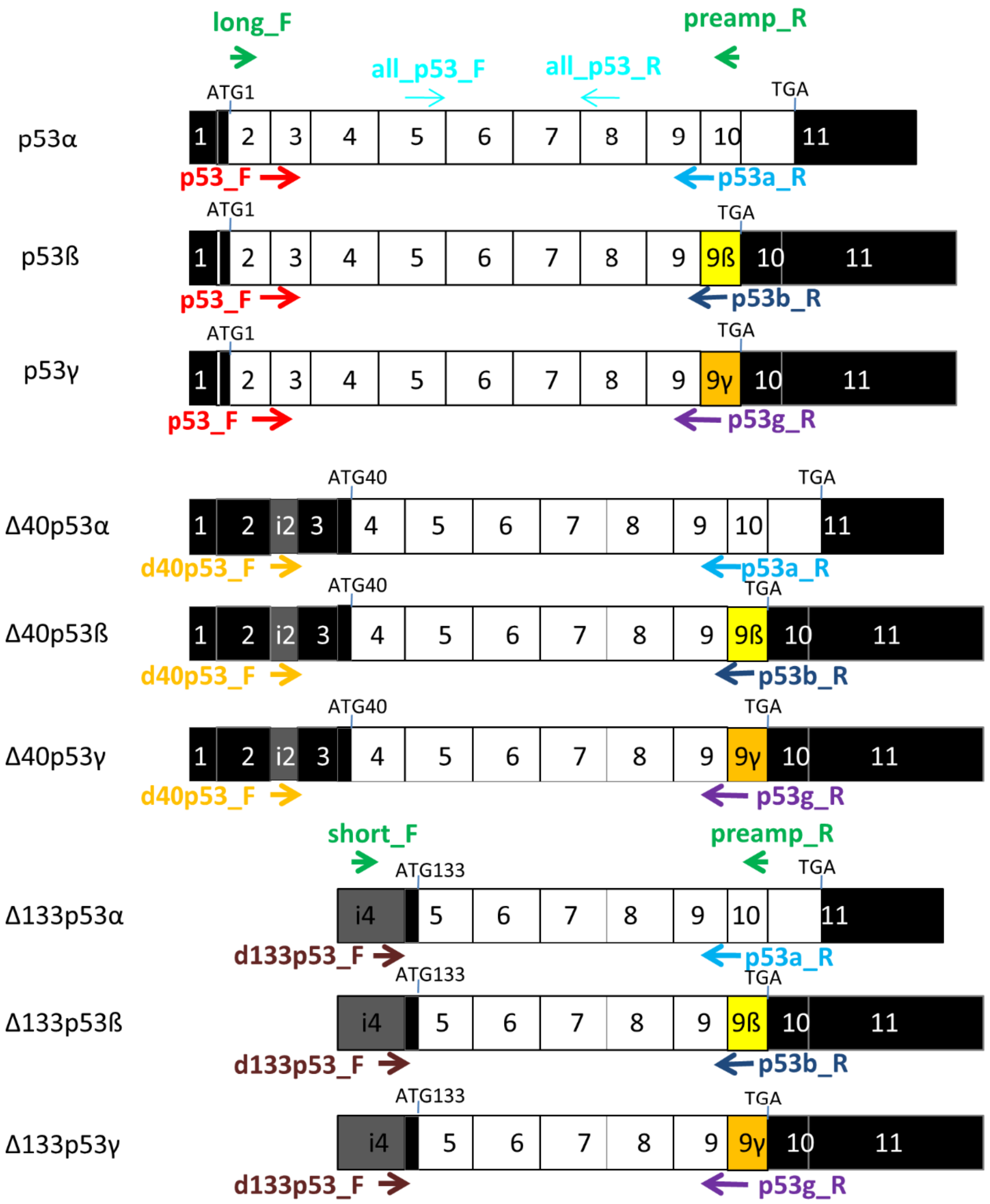

Supplementary Figure S3: Position of primers used to quantify the expression of nine different *TP53* mRNA isoforms. First, two nested PCR reactions were performed to distinguish "longer" full-length and Δ40 from "shorter" Δ133 isoforms. Thus pre-amplified cDNA templates were used for qPCR with combinations of three forward and three reverse primers. On both types of pre-amplified templates, a qPCR reaction for all *TP53* mRNA isoforms was also performed.

Supplementary Table S1: Number and percentage of samples with positive Western blot detection of tested proteins.

PROTEIN	HEALTHY TISSUE POSITIVITY (%)	TUMOR TISSUE POSITIVITY (%)
**p53α**	29/30 (90%)	30/30 (100%)
**p53Δ40α**	7/30 (23.3%)	11/30 (36.7%)
**p53Δ133α**	11/30 (36.7%)	25/30 (83.3%)
**p53Δ160α**	9/30 (30%)	22/30 (73.3%)
**p53β**	9/30 (30%)	11/30 (36.7%)
**p53Δ133β**	4/30 (13.3%)	7/30 (23.3%)
**TAp73α**	7/30 (23.3%)	16/30 (53.3%)
**ΔNp73α**	4/30 (13.3%)	14/30 (46.7%)
**TAp73β**	25/30 (83.3%)	24/30 (80%)
**ΔNp73β**	17/30 (56.7%)	9/30 (30%)
**GLI1FL**	3/30 (10%)	13/30 (43.3%)
**N'ΔGLI1**	5/30 (16.7%)	21/30 (70%)
**GLI2FL**	6/30 (20%)	7/30 (23.3%)
**GLI2ΔN**	3/30 (10%)	24/30 (80%)
**GLI3A**	21/30 (70%)	23/30 (76.7%)
**GLI3R**	19/30 (63.3%)	22/30 (73.3%)
**NME1**	28/30 (93.3%)	28/30 (93.3%)
**NME2**	27/30 (90%)	27/30 (90%)

Supplementary Table S2: Correlation table of gene expression in tumor samples. Significant correlations ($p < 0.05$) are marked in bold.

p53β	p53γ	Δ40p53α	Δ40p53β	Δ40p53γ	Δ133p53α	Δ133p53β	Δ133p53γ	TAp73	ΔNp73	*NME1*	*NME2*	*GLI1*	*GLI2*	*GLI3*	*PTCH1*		
0.22	0.21	**0.54**	-0.20	-0.35	-0.05	0.02	-0.06	-0.25	-0.13	0.15	-0.01	-0.08	-0.23	-0.20	0.04	ρ[†]	p53α
0.292	0.333	**0.007**	0.357	0.099	0.854	0.941	0.840	0.260	0.568	0.506	0.957	0.723	0.297	0.361	0.869	P	
	0.05	**0.44**	**0.45**	0.25	0.41	0.11	0.38	0.35	-0.08	0.34	0.25	-0.13	0.07	-0.06	-0.08	ρ	p53β
	0.826	**0.032**	**0.026**	0.246	0.119	0.674	0.164	0.100	0.727	0.109	0.246	0.559	0.768	0.802	0.730	P	
		0.37	**0.45**	**0.44**	-0.20	-0.33	-0.32	0.05	-0.21	0.21	-0.01	-0.15	0.05	-0.17	-0.29	ρ	p53γ
		0.087	**0.031**	**0.036**	0.467	0.209	0.267	0.823	0.357	0.366	0.982	0.529	0.827	0.471	0.209	P	
			0.22	0.12	0.14	-0.06	0.06	0.23	-0.01	0.34	0.11	**-0.43**	-0.17	0.16	-0.31	ρ	Δ40p53α
			0.300	0.582	0.602	0.823	0.830	0.299	0.962	0.120	0.619	**0.048**	0.450	0.478	0.165	P	
				**0.93**	0.17	0.10	0.13	**0.45**	-0.18	0.10	0.05	-0.04	0.27	0.10	-0.21	ρ	Δ40p53β
				**<0.0001**	0.520	0.715	0.657	**0.036**	0.434	0.658	0.832	0.859	0.218	0.647	0.347	P	
					-0.05	0.03	0.00	0.36	-0.21	-0.08	-0.10	-0.04	0.23	0.13	-0.34	ρ	Δ40p53γ
					0.863	0.903	0.990	0.099	0.352	0.729	0.655	0.856	0.301	0.566	0.128	P	
						**0.64**	**0.90**	0.31	-0.01	0.24	-0.10	**-0.66**	-0.31	-0.21	-0.08	ρ	Δ133p53α
						**0.007**	**<0.0001**	0.266	0.980	0.390	0.733	**0.007**	0.266	0.459	0.781	P	
							**0.87**	**0.67**	0.27	-0.03	0.09	-0.28	0.03	0.39	0.12	ρ	Δ133p53β
							**<0.0001**	**0.003**	0.305	0.903	0.729	0.273	0.911	0.125	0.639	P	
								**0.57**	0.15	0.11	0.01	-0.50	-0.13	0.24	-0.08	ρ	Δ133p53γ
								**0.027**	0.585	0.704	0.970	0.056	0.639	0.383	0.781	P	
									**0.38**	0.27	0.28	0.09	**0.39**	**0.43**	0.10	ρ	TAp73
									**0.031**	0.132	0.127	0.638	**0.030**	**0.013**	0.587	P	
										-0.07	0.16	0.04	0.01	**0.39**	**0.46**	ρ	ΔNp73
										0.717	0.369	0.842	0.975	**0.028**	**0.008**	P	
											**0.58**	-0.09	0.10	0.22	0.21	ρ	*NME1*
											**0.001**	0.635	0.602	0.235	0.248	P	
												0.17	**0.44**	0.26	**0.48**	ρ	*NME2*
												0.365	**0.012**	0.149	**0.005**	P	
													**0.67**	-0.11	0.33	ρ	*GLI1*
													**<0.0001**	0.561	0.062	P	
														0.12	0.32	ρ	*GLI2*
														0.517	0.079	P	
															0.15	ρ	*GLI3*
															0.412	P	

[†]ρ - Spearman rank correlation coefficient

Supplementary Table S3: Correlation table of protein expression in tumor samples. Significant correlations ($p < 0.05$) are marked in bold.

p53β	Δ40p53α	Δ133p53α	Δ133p53β	Δ160p53α	TAp73α	TAp73β	ΔNp73α	ΔNp73β	NME1	NME2	N'ΔGLI1	GLI1FL	GLI2ΔN	GLI2FL	GLI3A	GLI3R		
0.17	0.01	0.34	**-0.43**	**0.48**	**0.55**	**0.73**	**0.41**	0.34	0.28	0.24	0.32	**0.60**	0.28	-0.08	0.36	**0.62**	ρ†	p53α
0.379	0.969	0.064	**0.019**	**0.008**	**0.002**	**<0.0001**	**0.026**	0.071	0.134	0.194	0.082	**0.0004**	0.136	0.660	0.050	**3E-04**	P	
	-0.15	0.20	0.17	**0.39**	0.01	-0.14	-0.08	0.09	-0.10	-0.21	-0.05	-0.09	-0.04	0.09	-0.06	0.12	ρ	p53β
	0.446	0.283	0.378	**0.035**	0.961	0.453	0.670	0.637	0.611	0.273	0.793	0.639	0.848	0.639	0.735	0.527	P	
		0.33	-0.16	-0.11	-0.27	0.01	-0.12	0.12	0.08	0.31	0.20	0.20	0.10	-0.02	0.26	0.20	ρ	Δ40p53α
		0.077	0.400	0.561	0.150	0.957	0.543	0.531	0.671	0.093	0.298	0.301	0.608	0.938	0.168	0.297	P	
			**-0.53**	**0.40**	0.17	**0.40**	0.28	0.27	0.31	**0.41**	0.12	0.11	**0.45**	0.09	-0.07	0.22	ρ	Δ133p53α
			**0.002**	**0.030**	0.362	**0.029**	0.129	0.150	0.093	**0.027**	0.531	0.577	**0.012**	0.625	0.726	0.237	P	
				-0.26	**-0.43**	**-0.54**	**-0.41**	-0.15	-0.29	-0.29	-0.34	-0.15	-0.35	-0.13	-0.13	**-0.38**	ρ	Δ133p53β
				0.169	**0.018**	**0.002**	**0.026**	0.434	0.123	0.115	0.068	0.443	0.057	0.486	0.499	**0.039**	P	
					**0.48**	0.16	**0.46**	-0.25	0.25	0.26	0.19	**0.43**	0.24	-0.23	-0.03	0.21	ρ	Δ160p53α
					**0.008**	0.408	**0.010**	0.179	0.191	0.173	0.328	**0.017**	0.204	0.214	0.873	0.260	P	
						**0.61**	**0.52**	-0.14	0.23	0.32	**0.49**	**0.39**	0.27	0.11	**0.38**	0.19	ρ	TAp73α
						**0.0003**	**0.003**	0.477	0.217	0.087	**0.006**	**0.034**	0.149	0.551	**0.039**	0.324	P	
							0.35	0.27	0.25	0.34	**0.42**	**0.40**	0.20	0.17	0.28	**0.47**	ρ	TAp73β
							0.060	0.145	0.185	0.067	**0.020**	**0.031**	0.287	0.372	0.136	**0.009**	P	
								0.02	**0.38**	0.36	**0.47**	**0.52**	0.34	-0.22	0.13	0.08	ρ	ΔNp73α
								0.939	**0.040**	0.050	**0.010**	**0.003**	0.070	0.233	0.509	0.686	P	
									-0.32	-0.24	-0.02	0.18	-0.08	-0.18	0.07	0.24	ρ	ΔNp73β
									0.087	0.198	0.931	0.342	0.666	0.333	0.711	0.209	P	
										**0.79**	0.19	0.10	**0.69**	0.11	-0.01	0.16	ρ	NME1
										**<0.0001**	0.329	0.605	**<0.0001**	0.547	0.957	0.414	P	
											0.33	0.25	**0.59**	0.02	0.13	0.02	ρ	NME2
											0.080	0.186	**0.001**	0.909	0.501	0.918	P	
												**0.69**	0.18	-0.07	**0.48**	0.27	ρ	N'ΔGLI1
												**<0.0001**	0.353	0.724	**0.007**	0.154	P	
													0.14	**-0.45**	0.33	0.32	ρ	GLI1FL
													0.453	**0.012**	0.071	0.085	P	
														0.20	0.06	0.04	ρ	GLI2ΔN
														0.288	0.775	0.847	P	
															0.31	0.21	ρ	GLI2FL
															0.092	0.256	P	
																**0.38**	ρ	GLI3A
																**0.036**	P	

†ρ - Spearman rank correlation coefficient

Supplementary Table S4. Clinicopathological characteristics of samples (N=38).

**Characteristic**	**No. (%)**
**Gender**	
male	27 (71.1)
female	11 (28.9)
**Age** (years)	
median	66
range	39-87
≤70 y	28 (73.7)
>70 y	10 (26.3)
**Location of tissue sample**	
skin	13 (40.6)
lymph node	13 (40.6)
other	6 (18.8)
N.D.†	6
***BRAF* mutation status**	
negative	18 (47.4)
positive	20 (52.6)
***TP53* mutational status**	
negative	27 (90.0)
positive	3 (10.0)
N.D.	8
**No. of previous metastases**	
0	2 (5.6)
1	13 (36.1)
≥2	21 (58.3)
N.D.	2
**Subsequent metastases**	
absent	20 (55.6)
present	16 (44.4)
N.D.	2
**Radiation**	
no	18 (66.7)
yes	9 (33.3)
N.D.	11
**Chemotherapy**	
no	8 (34.8)
yes	15 (65.2)
N.D.	15
**Survival**	
alive	19 (50.0)
deceased	19 (50.0)

† N.D. - not determined

Supplementary Table S5: Sequences (5'–3' direction) of custom TaqMan Gene Expression primers and probes used for TAp73 and ΔNN'p73 gene expression analysis.

Gene	Forward primer	Reverse primer	Probe
TAp73	CCTCTGGAGCTCTCTGGAACC	TGGGCCATGACAGATGTAGTCA	CACCTACTTCGACCTTCCCCAGTCAAGC
ΔNN'p73	GCGCCTACCATGCTGTACGT	AGATTGAACTGGGCCGTGG	TGACCCCGCACGGCACCTC
TBP	CACGAACCACGGCACTGATT	TTTTCTTGCTGCCAGTCTGGAC	TGTGCACAGGAGCCAAGAGTGAAGA

Supplementary Table S6: Sequences of primers used for specific quantification of nine different N- and C-terminal isoforms of *TP53* mRNA by nested qPCR. The target regions for each set of primers are indicated.

Reaction	***TP53* mRNA isoform**	**Primer name**	**Targeted region**	**5'–3'sequence**
Pre-amplification PCR	"long"	long_F†	Ex2.1§	GTCACTGCCATGGAGGAGCCGCA
	"short"	short_F	Int4¶	TTCCAGTTGCTTTATCTGTTCACTTGT
		preamp_R‡	Ex10	CTTCCCAGCCTGGGCATCCTTG
qPCR	N-terminal			
	full-length	p53_F	Ex2/Ex3	AGACCTATGGAAACTACTTCCT
	Δ40	d40p53_F	Int2	GATCCATTGGAAGGGCAGGCC
	Δ133	d133p53_F	Int4	ACTCTGTCTCCTTCCTCTTCCTACAG
	C-terminal			
	α	p53a_R	Ex9/Ex10	CTCACGCCCACGGATCTGA
	β	p53b_R	Ex9β/Ex9	AAGCTGGTCTGGTCCTGAAGGGT
	γ	p53g_R	Ex9/Ex9γ	TCGTAAGTCAAGTAGCATCTGAAGG
	all *TP53* mRNA	all_p_53_F	Ex5	CCATCTACAAGCAGTCACAGCA
		all_p_53_R	Ex8	TTCTTGCGGAGATTCTCTTCCT

†F, forward; ‡R, reverse; §Ex, exon; ¶Int, intron

Supplementary Materials and Methods

Patients

All patients were treated at the Sestre milosrdnice University Hospital Center and clinical data were available. The study was approved by the Ethics Review Committee of Sestre milosrdnice UHC and the Bioethical committee of Ruđer Bošković Institute. Informed consent according to the World Medical Association Declaration of Helsinki was obtained from all patients. The tissues were collected during surgery, frozen immediately in dry ice and stored at -80°C until extraction. Survival time was measured from the date of surgery to the time of death or the last follow-up observation. The median follow-up of patients at the time of analysis was 16 months (range 1–38 months). The information on mortality was obtained from Croatian National Cancer Registry, Croatian Institute of Public Health.

RNA extraction, RT and qPCR analysis

Total RNA was extracted from 50 mg of frozen tissues using the TRIzol Reagent and purified on PureLink RNA Mini spin columns (all Thermo Fisher Scientific, USA) including on-filter DNase digestion (RNase-free DNase Set; Qiagen, Germany). From cell lines total RNA was extracted using the PureLink RNA Mini spin columns. RNA was subjected to reverse transcription to generate cDNA using High Capacity cDNA Reverse Transcription Kit (Thermo Fisher Scientific) according to the manufacturers' instructions.

qPCR analysis was performed in duplicate using TaqMan Gene Expression Master Mix and TaqMan Gene Expression Assays (all Thermo Fisher Scientific) on 100 ng of cDNA according to the manufacturers' instructions for *NME1* (Hs00264824_m1), *NME2* (Hs00897133_g1), *GLI1* (Hs01110766_m1), *GLI2* (Hs01119974_m1) and *GLI3* (Hs00609233_m1) genes, and ß-glucuronidase (*GUSB*; Hs00939627_m1) and TATA-binding protein (*TBP*; Hs00427620_m1) as reference genes. The qPCR analysis of *TP73* isoforms (TAp73 and ΔNN'p73) was performed in duplicate using TaqMan Gene Expression custom primers and TAMRA-labeled probes (Metabion, Germany) with *TBP* as reference gene (Supplementary Table S5), in 25 µL reaction mixture containing 125 ng of cDNA, 1 × TaqMan Gene Expression Master Mix (Thermo Fisher Scientific), forward and reverse primers (900 nM each) and labeled probe (250 nM). Gene expression analyses were performed according to protocol suggested by the manufacturer on the 7300 Real-Time PCR System (Thermo Fisher Scientific). Threshold cycle values were normalized to reference gene(s) (ΔCt) and relative expression was calculated using the $2^{-\Delta Ct}$ method.

To distinguish the three different N-terminal *TP53* isoforms, a nested qPCR approach was used. For longer N-terminal isoforms (full-length and Δ40), a new DNA template was initially made in pre-amplification PCR using long F and R primers with 25 ng of cDNA. For shorter Δ133 isoforms, a template was initially

made in pre-amplification step using short F and same R primer with 50 ng of cDNA. PCR was performed on 2720 Thermal Cycler (Thermo Fisher Scientific) in 25 µL reaction mix containing either 25 or 50 ng of cDNA, 1 × GoTaq Colorless Master Mix (Promega, USA), forward and reverse primers (400 nM each). PCR conditions for both pre-amplifications were: [95°C for 3 min (1 cycle)], [94°C for 30 s, 60°C for 40 s, 72°C for 1 min 30 s (35 cycles)] and finally [72°C for 7 min (1 cycle)]. The "long" pre-amplified templates were diluted 1:400, while the "short" was diluted 1:200.

For qPCR analysis of nine different *TP53* mRNA isoforms, combinations of three forward and three reverse primers were used (Supplementary Table S6). For full-length and Δ40 isoforms, p53_F and d40p53_F were used, respectively, both in combination with each of the three reverse primers (p53a_R, p53b_R and p53g_R) on "long" cDNA templates. For Δ133 isoforms, d133p53_F was used in combination with each of the three reverse primers on "short" templates. On both types of pre-amplified templates, a qPCR reaction for all *TP53* mRNA isoforms was also performed using all_p53_F and all_p53_R primers. qPCR was performed on the CFX96 Real-Time PCR Detection System (Bio-Rad, Hercules, CA, USA) in 10 µL reaction mix containing 1 × Takyon Low Rox SYBR MasterMix dTTP Blue (Eurogentec, Belgium), forward and reverse primers (400 nM each), 1 µL of diluted pre-amplified cDNA template and Milli-Q water. qPCR conditions were the following: [95°C for 3 min (1 cycle)], [95°C for 15 s, 63°C for 20 s, 72°C for 10 s (40 cycles)], and finally melting curve analysis from 72°C to 95°C with ramp of 0.5°C in 5 s. Threshold cycle values for nine *TP53* mRNA isoforms were normalized to appropriate all *TP53* mRNA variants' value (ΔCt) and relative expression was calculated using the $2^{-\Delta Ct}$ method.

Protein extraction and Western blot analysis

For 50 mg of tissues 0.8 mL of ice cold lysis buffer (50 mM Tris-HCl pH 8.0; 150 mM NaCl; 0.1% SDS; 0.5% sodium deoxycholate; 1% NP-40; 0.5 mM EDTA; 100 mM Na_3VO_4; 0.1% DTT) with protease inhibitors (Complete, Mini, EDTA- free; Roche Diagnostics, IN, USA) was added. Tissues were sonicated (3 mm probe, 4 × 15 sec) and centrifuged (10 min, 12500 rpm, +4°C). The whole cell lysates from cell lines were prepared in PBS with protease inhibitors followed by sonication (1 mm probe, 2 × 15 sec). Proteins were separated on 7% or 11% SDS-polyacrylamide gels. The antibodies used were as follows: sheep pantropic p53 antibody Sapu (1:5000); sheep anti-p53β (1:6000) and mouse anti-p53 (clone 421, 1:1000) all kindly provided by J.C. Bourdon; rabbit anti-p73 (EP436Y; Abcam, UK; 1:2000); rabbit anti-GLI1 (V812; Cell Signaling Technology, MA, USA; 1:1000), rabbit anti-GLI2 (ARP31885_T100; Aviva Systems Biology, CA, USA; 1:1000), rabbit anti-GLI3 (19949-1-AP; Proteintech, IL, USA; 1:600), rabbit anti-NME1 and NME2 (Nm23A&B 1:2000; kindly provided by I. Lascu and S. Volarević) and mouse anti-ß-actin (60008-1-1g, Proteintech 1:3000); secondary HRP-conjugated anti-mouse (GE Healthcare, UK;

1:3000), anti-sheep (Jackson ImmunoResearch Laboratories, UK; 1:10000), anti-rabbit (Cell Signaling Technology, 1:3000). Proteins were visualized using Western Lightning Chemiluminescence Reagent Plus (Perkin Elmer, MA, USA) or SuperSignal West Pico Chemiluminescent Substrate and SuperSignal West Femto Maximum Sensitivity Substrate (both Thermo Fisher Scientific).

***TP53* mutation analysis using FASAY method.** We were able to amplify the *TP53* region from 30/38 samples; the remaining 8 might have deletions within the *TP53* gene, or the RNA integrity did not allow further analyses. cDNAs from MCF7 (*TP53* wild-type) and MDA-MB-231 (*TP53* mutant) cells were used as controls. Unpurified PCR products were transfected along with HindIII/StuI (NewEngland Biolabs, MA, USA) double digested pRDI22 plasmid (LEU2) in yIG397 yeast reporter strain (ADE2) with the Lithium Acetate method (Raimondi et al., PLoS One. 2013; 8(7):e69152). Transformant colonies were selected on synthetic agar plates lacking leucine, but containing limiting concentration of adenine (5 mg/L). After three-night incubation at 30°C, yeast cells expressing a wild-type or mutant *TP53* generated respectively white or red colonies. According to a red colony frequency (RCF) score (see Knezović Florijan et al., Urol Oncol. 2019; 37(9):578.e1-578.e10 for more details), samples were sub-grouped as wild-type *TP53* (< 50% red colonies), heterozygous for *TP53* mutations (> 60%) or mutant *TP53* (almost 100%). Samples with an uncertain RCF (40-60%) were re-analyzed for a more precise analysis.

Statistical analysis

Since data of continuous variables didn't show normal distribution, which was tested with the D'Agostino-Pearson test, non-parametric statistical tests were used. To determine if there was a statistically significant difference in the expression between any two subgroups, the Mann-Whitney test was used for independent samples and the Wilcoxon test for paired samples. The Kruskal-Wallis test with a post-hoc test for pairwise comparison of subgroups according to Conover was used to determine statistically significant differences between multiple (>2) subgroups. Non-parametric Spearman rank correlation coefficient (ρ) was used to evaluate the correlation between continuous variables. Continuous variables were dichotomized into low and high categories by a median value or a criterion value associated with the highest Youden index obtained by the receiver operating characteristic (ROC) curve analyses. Pearson's chi-square test was used to assess the distribution and association of categorical variables. Overall survival (OS) curves were calculated with the Kaplan-Meier method and compared by the log-rank test. Two-tailed p-values less than 0.05 were considered statistically significant. Statistical analyses were performed using MedCalc, version 18.2.1 (MedCalc Software bvba, Belgium).
